# Reasons for the Formation of Non-Fibrous Inclusions When Preparing Basalt Fibers by the Duplex Method

**DOI:** 10.3390/ma13215033

**Published:** 2020-11-08

**Authors:** Anri Elbakian, Milan Sága, Boris Sentyakov, Ivan Kuric, Peter Kopas

**Affiliations:** 1 Department of Mechatronic Systems, Faculty of Quality Management, Kalashnikov Izhevsk State Technical University, Studencheskaya Street 7, 426069 Izhevsk, Russia; henry25@mail.ru (A.E.); sentyakov@inbox.ru (B.S.); 2 Department of Applied Mechanics, Faculty of Mechanical Engineering, University of Žilina, 010 26 Žilina, Slovakia; milan.saga@fstroj.uniza.sk; 3 Department of Automation and Production Systems, Faculty of Mechanical Engineering, University of Žilina, 010 26 Žilina, Slovakia; ivan.kuric@fstroj.uniza.sk

**Keywords:** fibrous materials, non-fibrous inclusions, basalt fiber, thermal insulation material, materials based on basalt fiber

## Abstract

Materials based on basalt fiber are widely used as thermal insulating material. These materials have a number of advantages, including their low thermal conductivity and fire resistance due to their natural composition. However, there is a significant drawback in that the material contain non-fibrous inclusions. The solution to this problem would significantly improve the working conditions of workers engaged in the production of materials from basalt fiber, as well as workers engaged in construction and installation works. In addition, the research will help to make completely new products, such as special fireproof paper and sterile medical materials. This article focuses on the reasons for the formation of non-fibrous inclusions in the production of this kind of material. The technology of producing canvases from superthin fiber in the duplex way is studied. The analysis of the production process is made. Certain technological and structural parameters of the influence on the formation of such inclusions are identified. Experiments are carried out and conclusions are drawn given formation of non-fibrous inclusions of various geometric shapes for various factors. A mathematical model of the process under consideration is built. The article draws conclusion on the application of these developments in the production cycle of creating materials based on basalt fiber.

## 1. Introduction

The task of industrial research is to bring new technology, material and the like into practice. Today, in addition, great emphasis is placed on environmental impacts, energy intensity of production, corrosion properties as well as other properties in the development process itself [1].

Canvases and other products from superthin basalt fiber are used in industrial and civil engineering for thermal insulation and sound insulation of buildings and structures as well as in the power industry for thermal insulation of steam pipelines, gas and electric heating devices. Also, they are used in mechanical engineering, automotive, aviation and shipbuilding industries in order to protect equipment from high or low temperature [2,3,4].

Longitudinal fibers made of basalt can be used in combination with a material other than the reinforcement of composites. It is this combination that brings unusual physical properties. These fibers thus bring high utility value at a low price. The main advantages of using basalt as a material in constructions include: relatively high mechanical resistance, resistance to abrasion, damage and flexibility; high acid resistance, high heat resistance and low flammability; low strength loss at high temperatures; higher tensile strength than steel fiber of the same dimensions; excellent thermal, acoustic and electrical insulating properties; high adhesion to polymer resins and rubber and environmentally friendly and non-toxic material. It can be argued that basalt fiber will in future be replaced by glass fiber composite materials, as they have similar physical properties. Thus, basalt fiber is predestined for use in the production of fire-resistant fabrics, brakes, brake pads and in the aerospace industry as a replacement for components, which are overpriced by the carbon content. These materials are gaining in importance in the military industry. As can be observed today, basalt-based composite materials have been used since the simplest components to the production of whole parts, for example in the civil industry. For the reasons described above, basalt fibers can be combined with various materials to achieve the desired properties [5,6].

However, due to the low quality of the feedstock, process violations and structural imperfections in the equipment, basalt fiber canvases often contain non-fibrous inclusions. The type of inclusions in basalt fiber canvases leads to an undesirable increase in the thermal conductivity of products as well as workers’ injuries.

**Figure 1:**
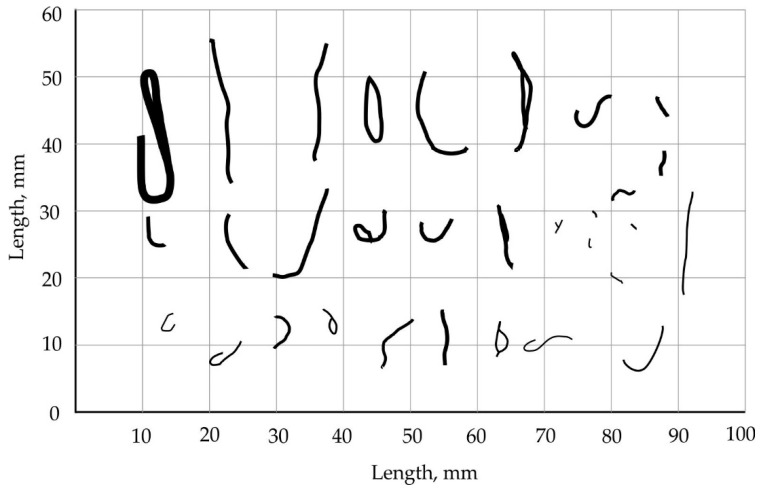
Varieties of non-fibrous inclusions.

A study on the formation of non-fibrous inclusions has shown that the shape and size of such inclusions are closely related to their underlying causes [7,8]. By eliminating these causes, it is possible to significantly improve the quality of basalt fiber products. Figure 1 demonstrates possible non-fibrous inclusions of various shapes and sizes. Their classification is given below [9].

## 2. Experimental Materials and Methods

The article considers duplex technology that is widely used in the production of superthin basalt fiber. The essence of this technology is to melt basalt chips in special furnaces at a temperature of 1400–1500 °C primary filaments with a diameter of 0.15–0.3 mm by pulling them from a spinneret feeder and further blowing of these filaments with a gas stream at a temperature of about 2000 °C and speed of 100–200 ms^−1^ with the formation of elementary staple fibers with a diameter of 1–3 microns which are deposited on a perforated conveyor in the form of a canvas.

**Figure 2:**
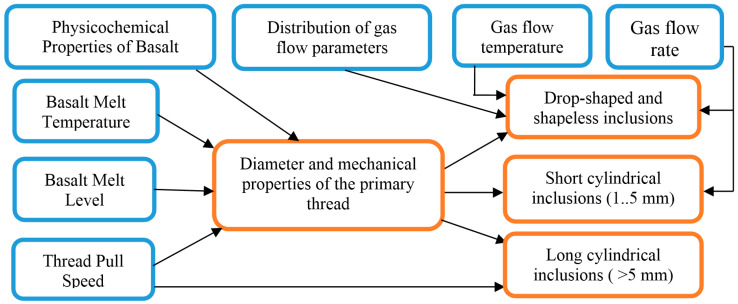
The influence of the physical parameters of the technological process on the formation of non-fibrous inclusions.

Based on several studies [10,11,12], it was shown that technological and design parameters affect the formation of non-fibrous inclusions. The size of the content of non-fibrous inclusions is influenced by the physical parameters of the technological process (Figure 2) and the parameters of the structural elements of the device.

**Figure 3:**
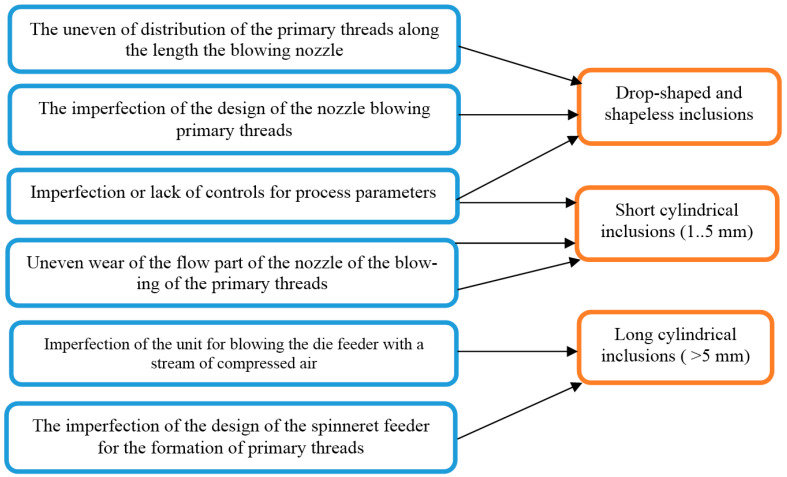
Technical disadvantages of equipment for obtaining basalt fiber using duplex technology.

The technical flaws of the equipment for producing basalt fiber by the duplex method and their connection with various types of non-fibrous inclusions are shown in Figure 3. The close arrangement of the filaments leads to their coalescence, the energy of the gas stream becomes insufficient for their melting and splitting, therefore, droplet-shaped formless inclusions are formed. Uneven wear of the blowing nozzle leads to uneven flow parameters along the length of the nozzle and the inequality of fiber formation conditions for the threads.

## 3. Conclusions of the Conditions of the Formation of Drop-Shaped and Loop-Like Non-Fibrous Inclusions

**Figure 4:**
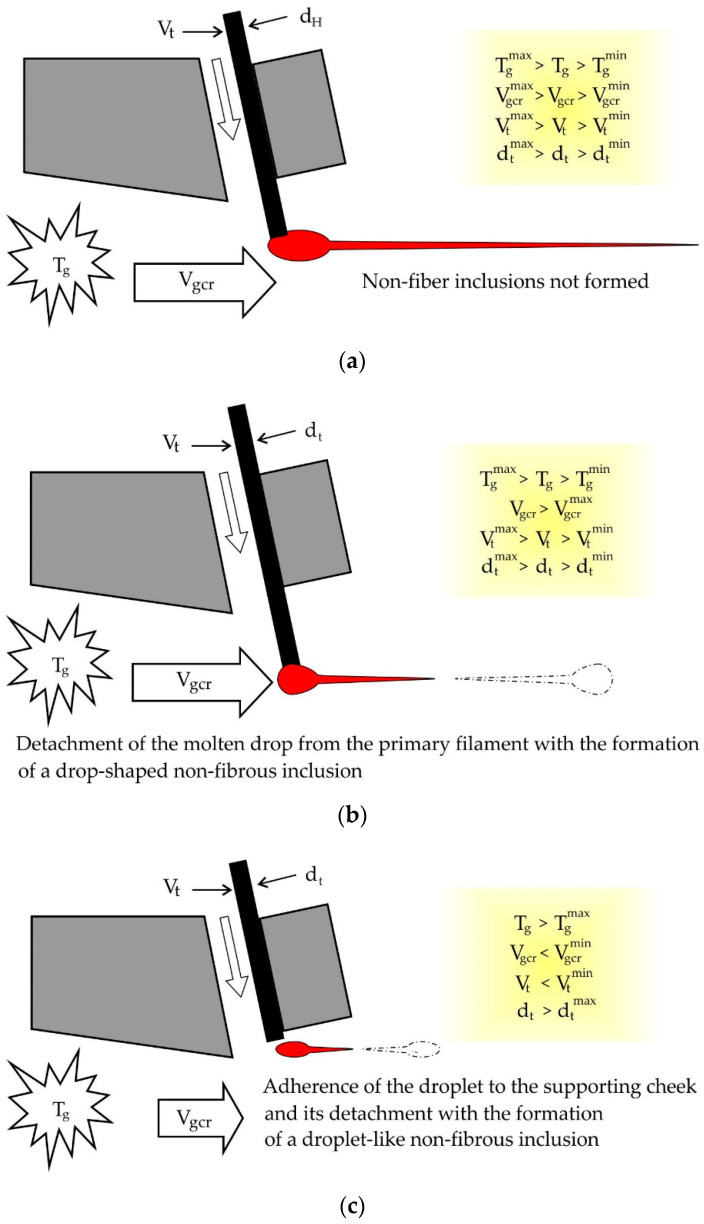
The process of formation of non-fibrous inclusions: (**a**) inclusions are not formed, (**b**,**c**) drop-like inclusions are formed.

Non-fiber inclusions close to the correct geometric drop-shaped form are formed by the detachment of the secondary drop of basalt melt formed at the end of the primary filament embedded in the gas stream, as shown in Figure 4. We may assume that formation process of such inclusions occurs near the upper edge of the slit nozzle inflator where the temperature gradient has the maximum value. In this case, it is possible to simulate the separation process of the drop from the primary thread as a solid, similar to Baluev and Stepanov’s work [13,14], where the distribution law of droplets in size when the liquid breaks off with the crests of the waves on the surface of the film, which is carried away by the gas stream.

To determine the patterns of formation of these inclusions, we consider the liquid particle formed during the breakaway process which after cooling turns into a drop-shaped non-fiber inclusion. It is affected by the power of inertia M_c_a, balanced by the pulling force of the flow F_flow_ and the force of the drop grip with the end of the primary thread F_clutch_:

M_c_a = F_flow_ + F_clutch_, (1)

The strength of the clutch is defined as the product of surface tension (on the perimeter of the drop’s interaction zone with the end of the primary strand:

F_clutch_ = πσd_t_, (2)

At the moment of separation, this force takes zero value. If we consider that the flowing speed of the drop is quite large and the strength of the breakaway is determined mainly by the magnitude of the flow rate of the gas mixture, it can be taken constant from the beginning of the break age to the end. Further Equation (2) follows:

M_c_a = F_clutch_, (3)

The beginning of the breakaway is characterized by the equality of breakaway and clutch forces, i.e., zero acceleration, so from Equation (1) taking into account Equation (3) we get a ratio:

πσd_t_ = π d_drop_^2^ C_x_ ρ_g_ V_med_^2^/8, (4)

from where the largest diameter of the drop-shaped inclusion is determined:

d_drop_ = (8σd_t_/C_x_ ρ_g_ V_med_^2^)^0.5^, (5)

where is C_x_—frontal resistance factor (C_x_ = 1, if you count the drop of spherical), ρ_g_—gas flow density, ns^2^/m^4^, V_med_—average gas flow speed, m/s.

Measuring the sizes of those with the correct geometric shape, drop-shaped inclusions, found in different batches of basalt fiber obtained at the industrial plant, have showed that their largest diameter varies from 1.2 d_t_ to 3.6 d_t_. From Equation (5) with d_t_ = 0.2 mm, V_med_ = 500 m/s and ρ_g_ = 0.124 ns^2^/m^4^ we calculate that the surface tension of the melt σ is between 1.12–10.1 N/m.

**Figure 5:**
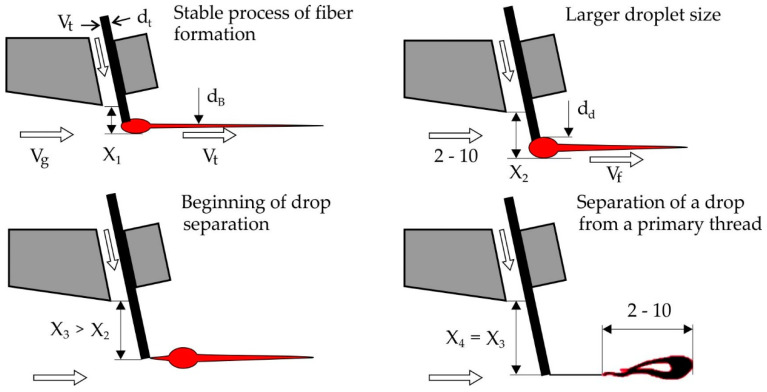
The process of forming loop-shaped non-fibrous inclusions.

Similarly, Figure 5 explain the formation of non-fibrous inclusions when pouring a secondary drop of molten basalt on the supporting cheek. In this case, the size of the non-fibrous inclusion, which most often has the wrong geometric, shape turn out to be larger and reach in a cross direction 3.8d_t_. This increase in their size is due to the fact that on the surface of the supporting cheek the flow rate is zero and the aerodynamic force of the detachment of inclusion decreases significantly. At the same time, by spreading the drop across the surface of the supporting cheek increases the strength of the clutch. The size of such a non-fibrous inclusion can be determined if you take advantage of G.N. Abramovich’s work which provides data on the distribution of speed in a semi-limited jet [15].

Having compiled the equation of the balance of the drop on the supporting cheek, it was obtained:

A_d_B_d_ = 8P_d_σC_cr_ρ_g_V_x_^2^, (6)

where is A_d_ and B_d_ is cross-size drop on the supporting cheek; P_d_ is perimeter of the drop zone with the supporting cheek; V_x_ is average flow rate with drop, m/s.

It has been proven that if you take the speed of the fiber equal to the speed of the gas flow V_b_ = V_g_, something out of the equation of inseparability:

(πd_t_^2^/4)V_t_ = (πd_f_^2^_/_4)_g_, (7)

The diameter of the fiber is determined by the formula:

d_f_ = d_t_(V_t_/V_g_)^0.5^, (8)

When a thread is inserted into the X_1_ stream, the size of the secondary drop does not exceed the diameter of the filament and no fibrous inclusions are formed.

Experimentally, heating the primary filament in the flame of the gas burner and measuring the diameter of the drop at its end with a micrometer, it is proven that with increasing the time t of finding the filament in the flame size drop secondary drop increases. The results of the experiment are presented in Table 1.

The amount of thread in flow is determined by the following formula:

X_1_ = tV_t_, (9)

Increase in the diameter of the drop at the end of the thread d_d_ reduces the strength of surface friction and the diameter of the fiber is initially increased without the formation of non-fibrous inclusions. Further increase V_t_ leads to an increase in d_drop_ and increase aerodynamic strength:

F_a_ = π d_drop_^2^C_x_ ρ_g_V_g_^2^/8K_e_, (10)

When F_a_ exceeds the grip of the drop with the thread:

F_clutch_ = πσd_t_, (11)

Begins to break the drop from the thread with a decrease in the screening factor K_e_ an increase in the diameter of the fiber and the formation on the drop of the second process. After the detachment of the non-fibrous inclusion from the thread under the influence of inertial forces, the right process unfolds against the direction of the flow and the inclusion takes the form of a loop.

## 4. Condition of Non-fibrous Inclusions in Destruction Threads under the Influence of Bending Stresses

**Figure 6:**
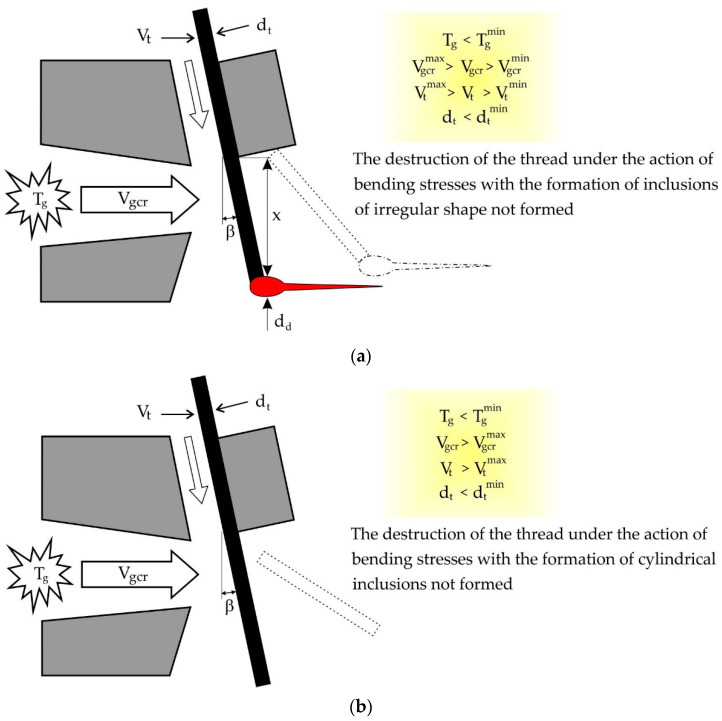
The process of formation of non-fibrous inclusions: (**a**) inclusions of irregular shape; (**b**) cylindrical inclusions.

One of the reasons for the formation of non-fibrous inclusions in basalt fiber in the production of its method of inflating the primary strands of basalt melt is the violation of the condition of the strength of the filaments when they bend under the influence of the gas stream, expiring from the nozzle inflate. The scheme of non-fibrous inclusions for this reason is demonstrated in Figure 6. This creates non-fibrous inclusions of two species. The first has an irregular geometric shape, which is formed with one line fragment of the primary thread 1 length 3 mm and on the other hand a drop-shaped element. They appear when the secondary drop of melt at the end of the thread is formed when the thread is inserted into the stream when the flow is not high enough. The second inclusions have the correct geometric shape in the form of fragments of primary strands of 2 to 8 mm long. They appear at a small diameter of the primary filament or at a low temperature of the gas flow, when the filament continues to move in the direction of its axis and does not have time to warm up to the melting temperature and, as in the first case, is destroyed by bending stresses acting from the gas flow side.

The condition for the destruction of the thread under the action of bending stresses is:

σ_i_ > [σ_i_], (12)

where is [σ_i_] = 50–90 MPa—the permissible voltage of the bend of the thread, the voltage of the bend of the thread, determined by the formula:

σ_i_ = M_i_/W_i_, (13)

where is W_i_ = πd_t_^3^/32—moment of resistance to the bend of the thread d_t_.

In the process of inflating the primary strands of basalt melt by gas stream, it is possible that at high speeds of pulling the filaments of the gas flow flowing from the slit nozzle and it is not enough to form a secondary drop at the end of the filament Melt. In this case, it is possible to break the thread under the influence of the bending moment and form a non-fibrous inclusion in the form of a cylindrical element of the correct geometric shape.

Assuming that the gas flow creates an evenly distributed q load on the protruding part of the thread, as shown in Figure 6, the maximum bending moment:

M_i_ = qX^2^/2, (14)

The distributed load on the surface of the thread will be determined by:

q = C_x_d_t_ρ_g_V_med_^2^ cosβ/2, (15)

where is C_x_ is frontal resistance ratio of the filament; ρ_g_ is gas flow density; V_med_ is average gas flow speed; β is angle between the direction of the filament and the perpendicular to the direction of the gas flow. From Equations (12)–(15) critical flight of the primary thread:

X_drop_ = (πd_t_^2^[σ_i_]/8 C_x_ ρ_g_ V_med_^2^ cosβ)^0.5^, (16)

By substituting in Equation (16) the values of the settings in it d_t_ = 0.15–0.3 mm; V_med_ = 400–600 m/s; β = 10°; ρ_g_ = 0.124 ns^2^/m^4^; C_xd_ = 0.05; C_x_ = 1; [σ_i_] = 50–90 MPa.

It is calculated that the critical departure of the thread, at which it is possible to destroy it is in the range of 2.1 to 6.8 mm. Taking into account the force of the flow to the drop, the critical departure of the primary thread is determined from the expression:

M_iq_ + M_iR_ = πd_t_^3^[σ_i_]/32, (17)

where M_iq_ is bending the moment under the influence of a distributed load on the cylindrical part of the thread, which is in the stream; M_iR_ is bending moment caused by the action of aerodynamic force on the secondary drop of the melt. By substituting in Equation (17) moments, we obtain:

X_t_^2^d_t_C_t_ρ_g_V_med_^2^ cosβ/2 + X_t_ π d_t_^2^C_x d_ ρ_g_V_med_^2^cosβ/8 = πd_t_^3^[σ_i_]/32, (18)

Indicate a = d_t_ C_x_ ρ_g_ V_med_^2^ cosβ/2; b = π d_t_^2^ C_x t_ ρ_g_ V_med_^2^ cosβ/8; c = πd_t_^3^[σ_i_]/32 and having solved the resulting square equation, the following expression is obtained:

X_t_ = (−b ± (b^2^ + 4ac)^0.5^)/2a, (19)

By substituting in Equation (19) the limits of known parameters, it is obtained that the maximum and minimum flight of the thread, in which to break the thread under the influence of the bending moment, acting on the side of the gas flow is, respectively 2.8 and 8.7 mm, which confirms the possibility of non-fibrous inclusions for this reason.

**Figure 7:**
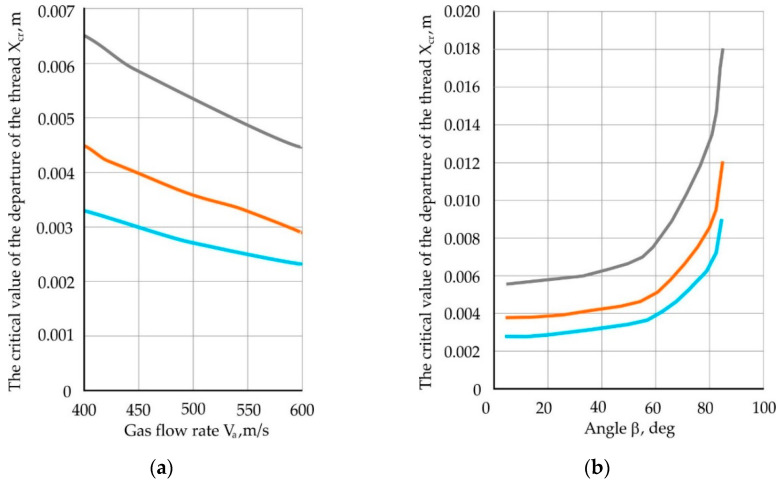
Dependence of the critical value: (**a**) of the filament departure on the gas flow rate at an input angle of the primary filament 10°; (**b**) of the take-off of the thread, on the angle β, at a gas flow velocity of 500 m/s.

Varying speed parameters V_g_ and the angleβ, you can see the dependence of a critical magnitude X_cr_ from these parameters. The resulting dependencies are presented on the graphs in Figure 7. They allow us to judge the extent to which the flow rate and the angle of the primary filament is inserted into the flow by the magnitude of the critical departure, which is important in the design of the basalt fiber plant and when assigning technological modes process.

## 5. The Formation of Cylindrical Non-Fibrous Inclusions When the Thread Loses Longitudinal Stability

With the reduction in the diameter of the threads, their longitudinal stability is lost in areas from the filler feeder to the drive rollers and from the rollers to the nozzle of the inflator, which leads to their mechanical destruction and the appearance of non-fiber inclusions up to several tens of millimeters. At the same time, if the thread loses stability after the drive rollers, the length of inclusions is within the distance to the nozzle inflator, which is about 100 mm and this does not cause a significant disruption of the process. If the loss of stability occurs on the site from the filler feeder to the drive rollers, the length of inclusions is several tens of millimeters, and this requires re-refueling the threads in the drive rollers. The condition, when the thread loses stability on the site from the drive rollers to the nozzle inflator and can occur its destruction, has the appearance:

F_fr_ > P_cr_, (20)

**Figure 8:**
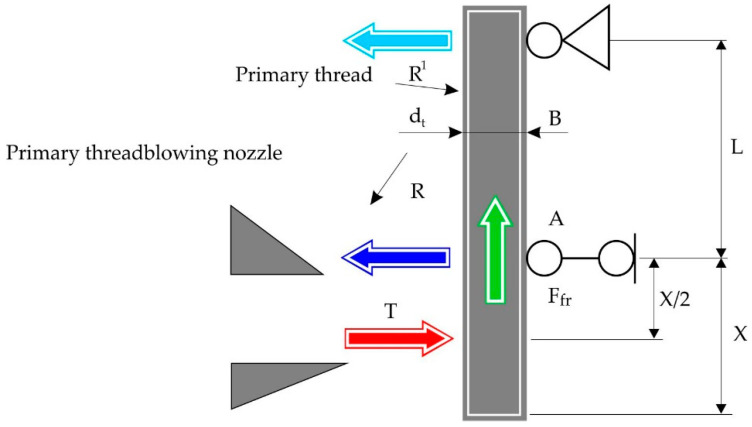
Conclusion of the stability condition of the primary thread.

The calculation scheme, which shows the forces of force, is shown in Figure 8. Loss of stability occurs under the influence of friction F_fr_ between the thread and the supporting cheek, pointing along the axis of the thread. The magnitude of the friction force is determined by a known formula:

F_fr_ = R·*f*_fr_, (21)

where *f*_fr_ is friction factor in the contact area of the basalt filament and the steel support cheek, defined experimentally. Average friction factor after 25 experiments was *f*_fr_ = 0.12 with a medium-square measurement error 0.01. Support reaction R determined from the assumption that the aerodynamic force of the gas flow on the part of the filament is attached in the middle part of the filament:

R = T (L + x/2)/L,(22)

The aerodynamic force acting on the thread is determined by the formula:

T = (ρv^2^/2) C_x_d_t_x, (23)

where ρ = 0.12–0.14 Kg/m^3^ is gas density; C_x_ = 1 is filament frontal resistance ratio; x is the size of the introduction of the thread into the stream, which begins the process of fiber formation and depends on the temperature and speed of the v flow and on the diameter of the thread d_t_.

Using Equation (23) to replace T in Equation (22), we get:

R = (ρv^2^/2) C_x_d_t_x (L + x/2)/L, (24)

Critical longitudinal force, in which a thread loses stability, is determined by the formula:

P_cr_ = π^2^EJ_min_/4L^2^, (25)

where is E = 0.45 × 10^5^ − 0.75 × 10^5^ MPa is basalt elasticity module; J_min_ is the smallest moment of thread section inertia:

J_min_ = πd_t_^4^/64, (26)

By substituting the value Equation (26) in Equation (25), the condition of loss of stability (20) after simplification takes the form:

128*f*_fr_ρv^2^C_x_ x L (L + x/2) > π^3^Ed_t_^3^, (27)

Neglecting in the condition Equation (8) the size of x/2, which is an order of magnitude smaller than L and indicating a size less set of constants and values that do not depend on the parameters of the device pulling threads through P:

P = 128ρv^2^C_x_/π^3^E, (28)

which for a functioning basalt fiber plant, at the above-mentioned limits of gas flow density and elasticity module, is within the limits of the P = 0.26 × 10^−6^ − 3.2 × 10^−6^, the condition of loss of stability takes the form of:

P*f*_fr_xL^2^/d_t_^3^ > 1, (29)

We introduce the concept of the criterion of longitudinal stability ξ, which is determined from Equation (9) and if ξ < 1, there are no non-fibrous inclusions:

ξ = P*f*_fr_xL^2^/d_t_^3^, (30)

**Figure 9:**
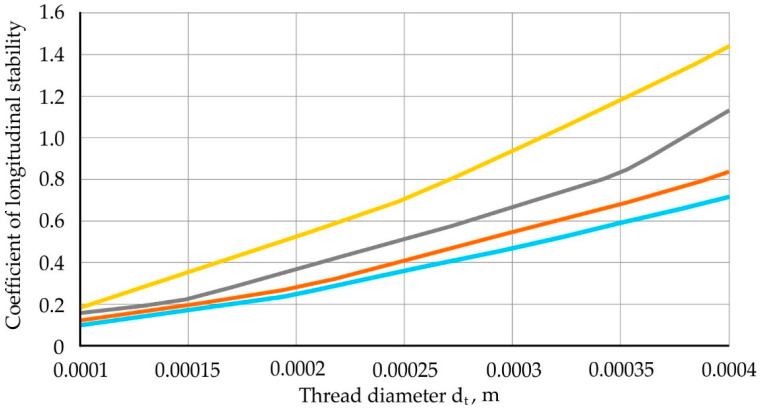
Dependence of the criterion of longitudinal stability of the thread ξ on the diameter of the thread d_t_ at L = 0.01 m and P = 0.26·10^−6^.

Longitudinal filament dependency charts ξ from the diameter of the thread d_t_. When thread is inserted into the stream x = 0.001; 0.002; 0.003 and 0.004 m when L = 0.01 m and P = 0.26 × 10^−6^ represented in Figure 9, which can be used to assign rational parameters of the process.

From the expression Equation (9) the critical value of the distance from the leading rollers to the nozzle of the inflator is determined:

L_cr_ = (d_t_^3^/P*f*_fr_x)^0.5^, (31)

**Figure 10:**
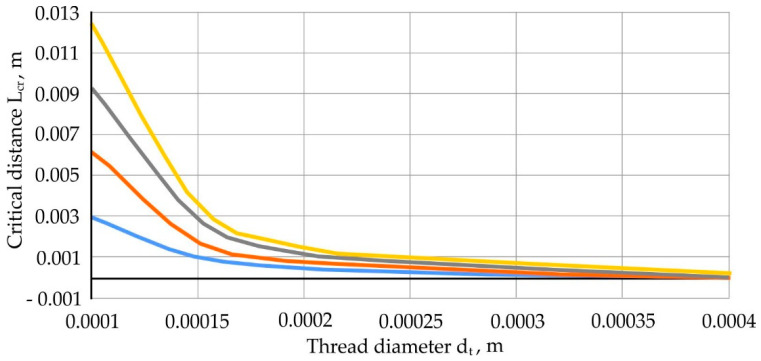
Dependence graphs of critical thread length L_cr_ from the diameter of the thread d_t_ at f_fr_ = 0.12 and P = 0.26 × 10^−6^.

Dependency charts L_cr_ from the diameter of the thread d_t_, when x = 0.001; 0.002; 0.003 and 0.004 m; *f*_fr_ = 0.12 and P = 0.26 × 10^−6^, presented in Figure 10.

For the ultimate case, when it is still possible to form a fiber, with a minimum diameter of the primary filament d_t_ = 0.1 mm and the maximum introduction of filament into the gas stream equal to the width of the slit nozzle inflator x = 6 mm, the critical distance from the nozzle inflator to the drive rollers, calculated from the condition Equation (29), is L_cr_ = 18 mm. At the current basalt fiber plant, this distance is 100 mm. This confirms the assumption that non-fibrous inclusions may be formed due to the loss of primary filament resistance. To eliminate this, it is recommended to install an additional support from the side of the slit nozzle, preventing the filament from bending and breaking.

## 6. Mathematical Model of the Process of Non-Fibrous Inclusions

The model consists of three systems of equations and inequalities describing the processes of obtaining primary strands of basalt melt, fiber formation and formation of non-fibrous inclusions. The first system of Equations (32)–(45) determines the patterns of primary filament formation and allows to calculate the diameter of the primary thread d_t_ and the performance of the process *Q* depending on the geometric size of the filler, level of basalt melt in the melting furnace H and its properties. The same system includes a condition Equation (35) that eliminates the phenomenon of exhaust resonance, in which there may be an uncontrollable change in the diameter of the thread, as well as the dependence of the magnitude of the introduction of the X thread in the stream from the time t and the speed of its pulling V_t_:

d_t_ = 2(Q/πV_t_)^0.5^, (32)

Q = πρ_b_gH R_f_^4/^8μ l_f_ + TR_f_^2/^8μ L_f_ − πp_3_R_f_^4/^8μ L_f_, (33)

0.436 ln (8μ_b_ L_f_ V_t_/ρ_b_g HR_f_^2^) R_f_^2^/L_f_ > L, (34)

L > 0.32 ln (8μ_b_ L_f_ V_t_/ρ_b_g HR_f_^2^) R_f_^2^/L_f_, (35)

X = tV_t_, (36)

The second system of Equations (36)–(39) determines the flow parameters in the fiber-forming zone-average velocities V_medium_ and flow temperatures T, and their distribution by section of the slit nozzle in the direction of the primary filament:

V_med_ = (G_b_ + G_g_)/BC, (37)

T = 966 − 0.482V_med_ + 0.003, (38)

V_x_ = V_med_ [1 − (2X/B)^3/2^]^2^, (39)

T_x_ = T [1 − (2X/B)^3/2^]^2^K_ej_, (40)

where G_b_ and G_g_ is air and gas consumption to produce flammable gas mixture, m^3^/s; B and C is cross-size slit nozzle, m; X is cross-section all-coordinate from the axis of the snot section symmetry, m; K_ej_ = 0.8–0.9 is the rate of decrease in gas flow temperature due to the ejection of air from the atmosphere.

Temperature dependence on flow speed Equation (37) obtained by Timofeev L.V. [16], and the distribution of speed is accepted by the law of G. Schlichting [17]. The speed and temperature of the flow in the real process is controlled by a change in gas and air pressure at the entrance to the mixing vortex chamber. The dependence of average speed and average temperature on gas and air pressure presented in Table 2.

The third system Equations (40)–(42) includes a set of inequalities that determine the conditions of non-fibrous inclusions: a condition Equation (39), in which no loop-like inclusions are formed, a condition Equation (40) in which no form is formed meek non-fibrous inclusions, a condition Equation (41), in which long non-fibrous inclusions are not formed:

d_d_^2^ C_x t_ ρ_g_V_g_^2^/8 K_t_ < σd_t_, (41)

X < (πd_t_^2^[σ_i_]/8C_x_ ρ_g_V_med_^2^ cosβ)^0.5^, (42)

(128ρv^2^C_x_/π^3^E) *f*_fr_X L^2^/d_t_^3^ < 1, (43)

The joint solution of these equations allows you to assign such parameters of the process, for example, the level of basalt melt, gas and air pressure in front of the mixing chamber, the consumption of compressed air to cool the filaments at the exit of the filer feeder and the speed of pulling threads at constant parameters of equipment—the size of filler, nozzle inflating the threads and properties of the original raw materials, in which there are no non-fiber inclusions. However, keep in mind that the performance of the equipment may not be as high.

## 7. Conclusions

The research will help to determine the causes of formation of non-fibrous inclusions in the production of basalt fiber by the duplex method. Technological and constructive parameters that affect the formation of non-fibrous inclusions are identified. The study has identified technological and constructive parameters that affect the formation of non-fibrous inclusions. We have drawn conclusions based on the formation of droplet-like, loop-shaped and cylindrical non-fibrous inclusions.

We have determined the main reasons for the formation of non-fibrous inclusions in the production of basalt fiber by the duplex method as a result of experimental studies of the process using existing industrial equipment and substantiated as a result of consideration of the physical processes of fiber formation and the production of primary basalt fibers. We have considered the possibility of reducing non-fibrous inclusions in canvases based on basalt fiber at the stage of blowing the primary strands of molten basalt for the formation of fibers. Using these data with the results of experiment [18] by the authors to reduce the presence of non-fibrous inclusions in already formed elementary canvases, it is possible to achieve maximum quality of basalt fiber products and their safer and more environmentally friendly production and use.

The results obtained in the study of the causes and patterns of the formation of non-fibrous inclusions during the interaction of the primary filament of basalt melt with a gas stream flowing from the slot nozzle of the blow-out made it possible to build a mathematical model of this process.

The algorithm of implementation provides for the formation of raw data - information about the properties of raw materials, geometric parameters of the filler feeder, the size of the slit nozzle inflating the primary strands, properties and the range of change in consumption gas mixture, the range of regulation of the speed of pulling threads. Then, we calculate the following parameters: the diameter of the threads, the length of the zone of its deformation, the amount of penetration of the thread into the stream, temperature and flow rate. The next stage is to check the reasons for the formation of non-fibrous inclusions and cyclic adjustment of the initial data and calculations of the main process parameters until the desired result is obtained that means a compromise between fiber quality and process performance. The main purpose of this mathematical model is to determine such process parameters at which the specified quality indicators of basalt fiber are fulfilled and the required process productivity is ensured.

After conducting additional experiments on the reasons why certain non-fibrous inclusions form, one can take a significant step towards reducing their concentration in basalt fiber canvases. A more detailed and accurate classification of non-fibrous inclusions will simplify the creation of process automation: indexing of non-fibrous inclusions in basalt fiber by means of an electronic base and a scanning device, adjustment of production technological parameters that affect the purity of the formed material, selection of technical parameters of the acoustic processing of primary canvases.

## Figures and Tables

**Table 1:** The dependence of the size of the drop on the heating time.

t, c	1	2	3	4	5
d_drop_/d_t_	1.17	1.36	1.53	1.78	1.82

**Table 2:** Speed and temperature dependence on gas and air pressure.

Gas Pressure P Gas, kPa	6	10	6	1	2
Air pressure Pair, kPa	10	10	10	8	5
Flow speed V_med_, m/s	435	515	426	348	275
Flow temperature T_med_, °C	1324	1512	1305	1162	1006
